# Design of Experiments for Matrix-Assisted Laser Desorption/Ionization of Amphiphilic Poly(Ethylene Oxide)-b-Polystyrene Block Copolymers

**DOI:** 10.3389/fchem.2021.740495

**Published:** 2021-09-09

**Authors:** Hélène Pizzala, Magalie Claeys-Bruno, Valérie Monnier, Michelle Sergent, Laurence Charles

**Affiliations:** ^1^ Aix Marseille Université, CNRS, UMR 7273, Institut de Chimie Radicalaire, Marseille, France; ^2^ Aix Marseille Université, UMR CNRS IRD 7263, Institut Méditerranéen de Biodiversité Marine et Continentale, Marseille, France; ^3^ Aix Marseille Université, CNRS, Centrale Marseille, FR 1739, Fédérations des Sciences Chimiques, Marseille, France

**Keywords:** MALDI, design of experiments, QSAR, space filling design, mass spectrometry, amphiphilic copolymers

## Abstract

Matrix-assisted laser/desorption ionization (MALDI) has become a very popular ionization technique for mass spectrometry of synthetic polymers because it allows high throughput analysis of low amounts of sample while avoiding the complexity introduced by extensive multiple charging of electrospray ionization. Yet, fundamental mechanisms underlying this ionization process are not fully understood, so development of sample preparation methods remains empirical. Reliable prediction for the optimal matrix/analyte/salt system is indeed still not possible for homopolymers and it becomes even more challenging in the case of amphiphilic block copolymers where conditions dictated by one block are not compatible with MALDI requirements of the second block. In order to perform MALDI of copolymers composed of poly (ethylene oxide) (PEO) and polystyrene (PS) blocks, it was postulated here that experimental conditions suitable for both species would also be successful for PEO-*b*-PS. Accordingly, designs of experiments based on Quantitative Structure Activity Relationship (QSAR) analysis were first implemented, studying the influence of 19 matrices and 26 salts on the laser fluence requested for successful MALDI. This analysis first permitted to highlight correlations between the investigated 10 descriptors of matrices and salts and the analytical response, and then to construct models that permits reliable predictions of matrix/salt couples to be used for one or the other homopolymer. Selected couples were then used for MALDI of a PEO-*b*-PS copolymer but no general trend was observed: experimental conditions expected to work often failed whereas ionic adducts of the copolymer were clearly detected with some matrix/salt systems that were shown to badly perform for constituting homopolymers. Overall, this rules out the working assumption stating that the MALDI behavior of chains composed of PEO and PS segments should combine the behavior of the two polymeric species. Yet, although requiring a dedicated design of experiments, MALDI of the amphiphilic PEO-*b*-PS copolymer was achieved for the first time.

## Introduction

Matrix-assisted laser/desorption ionization (MALDI) (Karas and Hillenkamp, 1988; Tanaka et al., 1988) is a technique allowing production of gas phase ions upon laser irradiation of a solid mixture of non-volatile analytes embedded in matrix molecules. It has become a very popular ionization technique for mass spectrometry of macromolecules because it allows high throughput analysis of low amounts of sample. However, fundamental mechanisms underlying this ionization process are still not fully understood, which is particularly detrimental to the development of MS methods for analytes exhibiting a great structural variety such as synthetic polymers. Sample preparation, that is, proper matrix selection as well as solid-state organization, is known to be a key issue in the success of MALDI-MS analyses. Typically, MALDI samples are obtained after solvent evaporation of a matrix/analyte mixture, and although no longer present in the solid sample, the nature of solvents used to prepare individual solutions has critical impacts on sample homogeneity and hence on MALDI-MS data quality (Yalcin et al., 1998). The matrix should also fulfill different criteria, some of which are clearly defined (strong absorptivity at the employed laser wavelength or good vacuum stability) whereas other requirements, such as a good miscibility of the matrix with the analyte in the solid state, are not easily related to physico-chemical parameters of the matrix. In the particular case of synthetic polymers that mostly ionize *via* cation adduction, the solid mixture to be laser-irradiated should also contain a salt. Once the cation is chosen based on the nature and size of polymeric chains, the nature of the counter-anion often influences salt solubility (and hence the amount of available cations) depending on the solvent selected for sample preparation. Moreover, ionization yield of synthetic polymers subjected to MALDI is very sensitive to relative molar concentration of components in the ternary mixture. The task becomes even more challenging when dealing with copolymers composed of blocks with different chemical properties that may dictate incompatible experimental conditions for each segment. Overall, as long as the role of most influential parameters controlling the MALDI process is not clearly identified, accurate prediction for the optimal matrix/polymer/salt system is not possible and development of sample preparation methods remains empirical, either starting from published protocols that were shown to work for a given polymeric system (*NIST Synthetic Polymer MALDI Recipes Database*) or using a trial and error approach.

To gain insights in the MALDI process, morphology and molecular interactions within MALDI samples have been studied using a variety of techniques operating on solid state analytes such as scanning electron microscopy (Doktycz et al., 1991; Horneffer et al., 2003), time-of-flight secondary ion mass spectrometry (Hanton et al., 1999), crystallography (Mele and Malpezzi, 2000), MS imaging (Hanton et al., 2008) or solid state nuclear magnetic resonance (Pizzala et al., 2009; Major et al., 2012; Pizzala et al., 2021). Although providing useful new information, reported findings could not be clearly related to molecular properties of components in the solid mixture, and hence could not allow construction of predictive rules. Alternatively, more global approaches based on design of experiments (DoE) can be developed to take into account not only the variety of parameters that may actually be involved in the MALDI process but also their synergistic effects. The first step towards fundamental understanding of MALDI indeed implies to clearly identify which parameters are critical to the process, their respective influence as well as their interaction. Validation of these parameters typically consists of developing a model which predictive character can be experimentally verified. In mass spectrometry, the use of DoE is an emerging trend for simultaneous optimization of numerous factors (Hecht et al., 2016). Focusing on the field of MALDI-MS for synthetic polymers, different approaches have been reported. Wetzel et al. used fractional factorial design to study the effects of five instrumental parameters for different mixtures of polystyrene (PS) with dithranol or all-*trans*-retinoic acid as the matrix (Wetzel et al., 2006). While detector voltage and delay time were the most influential parameters for PS when mixed with all-*trans*-retinoic acid, laser energy was a supplemental factor to be considered when using dithranol as the matrix. Full factorial design was used by Brandt et al. to study effect of molar mixing ratio of several synthetic polymers [PS, poly(dimethylsiloxane), poly(ethylene glycol) and poly(methylmethacrylate)], using eight frequently employed matrices, five salts and thirteen different solvents (Brandt et al., 2010). The optimal ternary mixture composition was found to highly depend on the studied combination matrix/polymer/solvent used. The same group then focused on PS to build a predictive model based on partial least square regression that enables appropriate matrix/salt/solvent combinations to be defined from a few experiments (Brandt and Ehmann, 2010). A general linear model was successfully employed by Badia et al. to optimize MALDI conditions for poly(ethylene terephthalate) (Badia et al., 2011b) and for poly(lactide) (Badia et al., 2011a). The quality assessment method developed by Kooijman et al. for unsupervised quantitation of sample preparation quality was based on eight parameters (such as number and intensity of detected peaks, angular and radial signal distribution) to describe spots imaged by MALDI (Kooijman et al., 2016; Kooijman et al., 2017). The method permitted to select the best sample preparation parameters to be employed for MALDI of PEG, PMMA and polytetrahydrofuran, which were found to be highly polymer-dependent.

Here, we have evaluated the performance of a new approach, using space filling design (Santner et al., 2003; Fang et al., 2006), to construct predictive models aimed at identifying optimal conditions for MALDI-MS of PS and PEG, in order to find suitable conditions to be used when these polymeric species are part of amphiphilic PEO-*b*-PS macromolecules, assumed to combine the behavior of each segment in MALDI. In order to remove any contributing roles of the solvent that introduce additional complexity (Weidner et al., 2011), solvent-free sample preparation (Skelton et al., 2000; Trimpin et al., 2001) was used to investigate 19 matrices combined with 26 cationization agents, and considering that the MALDI process is most efficient when requiring the lowest laser fluence.

## Materials and Methods

### Chemicals

Polystyrene (PS) sample (*M*_n_ 2,000 g mol^−1^) was from Fluka (Buchs, Switzerland), the poly(ethylene glycol) (PEG) sample (*M*_n_ 4,000 g mol^−1^) was from Sigma (St Louis, MO) while the PEO-*b*-PS block copolymer (*M*_n_ 1,800 g mol^−1^ for the PEO block, *M*_n_ 1,600 g mol^−1^ for the PS block) was purchased from Polymer Service GmbH (Altdorf, Germany). The following 19 matrices from Sigma were considered: 1,8,9-anthracenetriol (Dith), 2′,4′,6′-trihydroxyacetophenone (THAP), 2-(4′-hydroxybenzeneazo) benzoic acid (HABA), 3-hydroxypyridine-2-carboxylic acid (HPA), 2,3-dihydroxybenzoic acid (2,3-DHB), 2,4-dihydroxybenzoic acid (2,4-DHB), 2,5-dihydroxybenzoic acid (2,5-DHB), 2,6-dihydroxybenzoic acid (2,6-DHB), 2,5-dihydroxy-*p*-benzoquinone (DHBQ), 2-mercaptobenzothiazole (MBT), 3,5-dimethoxy-4-hydroxycinnamic acid (sinapinic acid, SA), *trans*-indoleacrylic acid (IAA), 3-methoxy-4-hydroxycinnamic acid (ferulic acid, FA), 5-chloro-2-mercaptobenzothiazole (CMBT), 5-chlorosalicylic acid (5-CSA), 9-nitroanthracene (9-NA), 9H-pyrido [3,4-b]indole (norharmane, NOR), anthracene-9-carboxylic acid (9-ACA), and α-cyano-4-hydroxycinnamic acid (HCCA). Salts used as cationizing agents were 16 alkali halides (combining lithium, sodium, potassium and rubidium to fluoride, chloride, bromide and iodide), 5 silver salts (AgF, AgCl, AgBr, AgI, and AgNO_3_) and 5 copper salts (Cu Cl, CuI, CuBr, CuCl_2_, and Cu(NO_3_)_2_) obtained from Sigma. Methanol used to prepare matrix solution subjected to fluorescence and UV-Vis experiments was from SDS (Peypin, France).

### MALDI Mass Spectrometry

All MALDI samples were prepared in solvent-free conditions according to the vortex method (Hanton & Parees, 2005). Prior mixing, all chemicals and materials were stored in a glove box operated at 20°C and relative humidity of 65%: these specific conditions permit capture of atmospheric water molecules by all components, and not by the most hygroscopic ones only (Major et al., 2012). The three solid components (matrix, polymer, salt) were introduced in a 10 ml poly(propylene) tube containing six stainless steel balls (3 mm diameter) (VWR International, West Chester, PA). Matrix/salt molar ratio was 50:10 while the amount of polymer was calculated so as to reach matrix/monomer ratio of about 1:1. Total amount of ternary mixtures was about 20–40 mg. Once capped, the tube was taken out of the glove box and held on a vortex mixer (ThermoFisher Scientific, Waltham, MA) for grinding of the content at a 2,400 min^−1^ frequency for 16 min, as optimized from a previous study (Major et al., 2012). After mixing, a few grains of the sample were applied onto the MALDI target with a spatula to form a thin layer. MALDI-TOF MS experiments were carried out using a Bruker Autoflex instrument (Bruker Daltonics, Leipzig, Germany) equipped with a nitrogen laser emitting at 337 nm, a single-stage pulsed ion extraction source, and dual microchannel plate detectors. Data acquisition was performed in reflectron mode and, besides laser fluence, all instrumental parameters were kept constant: pulse frequency: 10 Hz; accelerating voltage: +19 kV; extraction delay time: 100 ns. Each sample was prepared twice and deposited in quadruplicate on the MALDI plate. Mass spectra were recorded from each deposit after 100 laser shots. The working laser fluence was arbitrarily defined just above (2%) the fluence threshold. FlexControl software version 2.2 (Bruker Daltonics) was used for instrument control and data acquisition, and FlexAnalysis software version 2.2 (Bruker Daltonics) for data processing.

### Other Measurements

Additional experiments or calculations had to be performed to obtain matrix descriptors that were not available in the literature. Relative Fluorescence Intensity (RFI) was measured with a microplate spectrofluorimeter (Infinite 200, TECAN, Männedorf, Switzerland) from matrix solution in methanol (3.10^−3^ mol L^−1^) with excitation at 337 nm. Molar absorption coefficients were measured at 337 nm from matrix solution in methanol (1.10^−4^ mol L^−1^) on a Shimadzu UV-Visible spectrophotometer. Theoretical calculations were performed to determine dipole moments and ionization energies, with geometry optimization using a DFT B3LYP method (as implemented in Gaussian) (Frisch et al., 2004) adopting 6–311 g (d,p) basis set and MP2 and ROMP2 levels, respectively. Proton affinities of some matrices were obtained using a DFT PBE1PBE method with a 6–31 + g(2d,2p) basis set.

### Design of Experiments

Experimental designs are mathematical and statistical techniques allowing the organization of experiments. These planned designs lead to an optimal quality of the information and also to a reduction of the number of experiments. More particularly, Space Filling Designs were considered here in order to spread experimental points evenly throughout the domain of interest described by the 10 descriptors. Designs of experiments were constructed using WSP algorithm (Santiago et al., 2012) which selects points to be tested among the set of 494 candidate points. The modeling was performed using stepwise regression (Hocking, 1976; Draper and Smith, 1981) from a full degree 2 model by selecting predictive variables. At each step of the model construction, coefficients to be included or excluded were tested with F-tests.

## Results and Discussion

### Design of Experiments

Descriptors selected for MALDI matrices (X_1_–X_7_ in Table 1) are molecular parameters acknowledged to play effective roles in the MALDI process, such as absorption coefficient at the employed laser wavelength (ε_337_, L mol^−1^ cm^−1^), ionization energy (IE, eV), proton affinity (PA, kJ mol^−1^) used instead of the less documented cation affinity, pKa and relative fluorescence intensity at 337 nm (RFI_337_). Supplemental features such as molecular weight (MW, g mol^−1^) or dipole moment (ρ, Debye) were also considered. The three descriptors used to characterize cationizing agents are the radius (in pm) of their constitutive cation and anion, and their bond energy (kJ mol^−1^), respectively defined as X_8_, X_9_, and X_10_ in Table 2. Some of these descriptors, such as pKa (X_2_), ε_337_ (X_4_), RFI_337_ (X_5_), ionization energy of the matrix (X_6_) and bond energy of salts (X_10_), have been modified using a Log transformation in order to obtain a more uniform variation range. The main computed Y response was the laser fluence required to obtain MALDI signals. Doing so, we consider that optimal ternary mixtures are characterized by low laser fluence. In contrast, poorly efficient sample compositions are identified by the need for high energy to be supplied to the solid substrate in order to record MALDI signals. More precisely, Y corresponds to the lowest laser fluence enabling to measure intensity of at least 50 counts for the oligomer at the maximum of the polymer distribution when recording mass spectra after 100 laser shots. This response is expressed as a percentage of the total laser power and was experimentally varied between 20 and 75%. When no signal was observed once reaching this upper 75% limit, samples were arbitrarily assigned a 100% fluence. MALDI experiments were also considered as unsuccessful (hence designated by a 100% laser fluence) when displaying signals that do not correspond to the expected oligomers adducted with the cation from the supplemented salt. Indeed, sodium is a common matrix pollutant which was shown to efficiently compete for PS or PEG cationization in MALDI, even when present at concentration levels far below that of the added salt (Pizzala et al., 2021).

**TABLE 1:** Parameters selected as descriptors for matrices.

Matrix	X_1_ MW (g mol^−1^)	X_2_ pKa^*^ ^or^ ^#^	X_3_ ρ^#^ (Debye)	X_4_ ε_337_ ^§^ (L mol^−1^ cm^−1^)	X_5_ RFI_337_ ^§^	X_6_ IE^#^ (eV)	X_7_ PA^*^ ^or^ ^#^ (kJ mol^−1^)
2,3-DHB	154.12	2.94^[a]^	2.04	1,620	391	8.43	851^[f]^
2,4-DHB	154.12	3.29^[a]^	0.88	210	69	8.76	863^[f]^
2,5-DHB	154.12	2.97	2.78	3,990	8,008	8.23	855.78^[f]^
2,6-DHB	154.12	1.30^[a]^	1.73	1,490	236	8.35	864^[f]^
5-CSA	172.57	2.6	1.03	1,200	12,122	8.68	802.04
9-ACA	222.24	3.65	1.53	2,930	12,920	7.38	821.31
9-NA	223.23	43	4.01	2,950	663.36	7.64	875.32
CMBT	201.70	6.8	1.49	15,250	48	8.64	902.37
DHBQ	140.09	2.95^[a]^	0	410	97	9.48	791.97
Dith	226.23	7.16	4.22	6,720	312	8.22	885.51^[g]^
FA	194.18	4.04^[b]^	2.35	13,040	1,227	7.89	879^[h]^
HABA	242.23	3.57	4.93	17,731	31	3.82	949.98^[g]^
HCCA	189.17	2.6	2.65	24,730	59	8.48	841.55^[g]^
HPA	139.11	3.9^[c]^	4.55	680	3,216	9.46	898.49^[g]^
IAA	187.19	4.59	4.40	16,800	280	7.76	893.88^[g]^
MBT	167.25	6.93^[d]^	0.97	6,920	60	8.54	889.74^[g]^
NOR	168.19	6.8^[e]^	3.06	4,970	46,809	7.81	875.52^[g]^
SA	224.21	3.98^[b]^	4.52	14,900	2,159	7.91	875.88^[g]^
THAP	168.15	7.76	3.44	1,729	167	8.45	893.04^[g]^

* From the literature as referenced hereafter.

# Calculated values.

§ Experimental values.

a (Schiller et al., 2007).

b (Remily-Wood et al., 2009).

c (Chou et al., 1999).

d (Woods et al., 2000).

e (De Andres et al., 2010).

f (Mormann et al., 2000).

g (Mirza et al., 2004).

h (Jorgensen et al., 1998).

**TABLE 2:** Parameters selected as descriptors for cationizing agents.

						
X_8_, cation radius (pm)^[a]^				Li	Na	K	Rb	Cu		Ag	
				128	166	203	220	132		145	
X_9_, anion radius (pm)^[a]^				F	Cl	Br	I	NO_3_			
				64	99	121	140	200			
X_10_, bond energy (kJ mol–1)^[b]^ of cationizing agents											
LiF	1,030	NaF	910	KF	808	RbF	774	AgF	953	CuCl	992
LiCl	834	NaCl	769	KCl	701	RbCl	680	AgCl	910	CuBr	969
LiBr	788	NaBr	732	KBr	671	RbBr	651	AgBr	897	CuI	948
LiI	730	NaI	682	KI	632	RbI	617	AgI	881	CuCl_2_	2,774
								AgNO_3_	820	Cu(NO_3_)_2_	2,739

a As referenced for covalent radii.(*Cambridge Crystallographic Data Center* *www.ccdc.cam.ac.uk/products/csd/radii*).

b As reported in referenced handbook (Lide, 2008).

Combining 19 matrices with 26 salts leads to 494 matrix/salt couples characterized by 10 descriptors. Classically in QSAR study (Todeschini et al., 2020), a model is postulated and a subset of points is selected in order to calculate the estimations of the coefficients of this model. In our case, it is obvious that a degree 1 model is not sufficient (due to non-linear phenomenon) to model the behavior. Yet, a complete degree 2 model would need a very high number of experiments (at least 66 experiments). Alternatively, we propose a new approach which does not require postulation of a model but, instead, consists of selecting points according to a uniformity criterion. Among the 494 candidates, a subset of 25 points distributed as uniformly as possible in the space of the 10 descriptors was selected, using the WSP algorithm (Santiago et al., 2012) which guarantees a good uniformity of the points (Santiago et al., 2012; Beal et al., 2014). At the end of the experimentation, different statistical treatments were performed, such as stepwise regression to consider the most significant interaction terms or square terms.

### QSAR Study of PS

**FIGURE 1:**
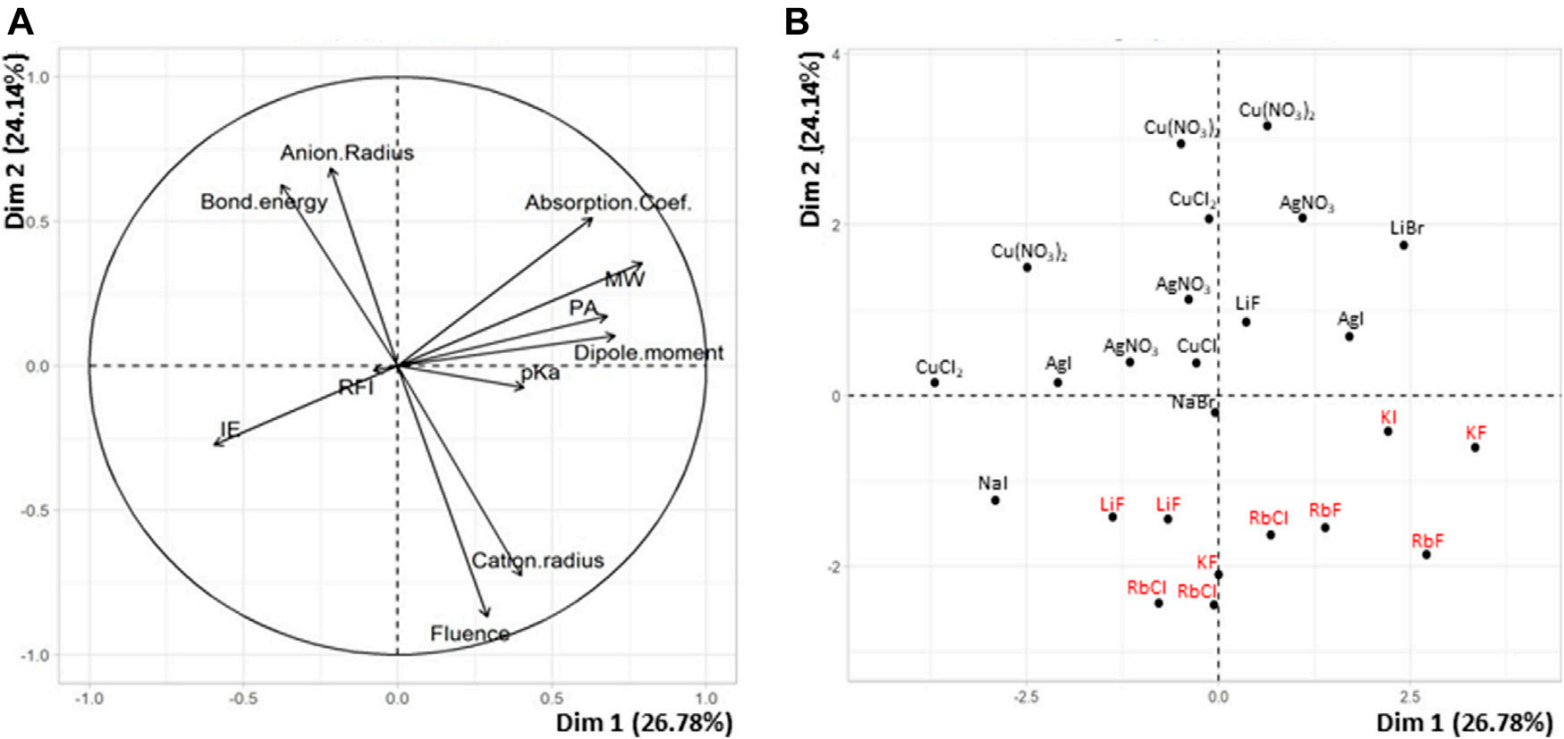
PCA plots of **(A)** variables and **(B)** individuals for the 25 matrix/salt couples tested for MALDI of PS.

Results obtained when testing the selected 25 matrix/salt couples for MALDI of PS are reported in Table 3. Before performing quantitative treatments to establish the variation of the fluence threshold as a function of the 10 descriptors, multidimensional descriptive analyses were performed to identify any general trends. Principal component analysis (PCA) was carried out for these 25 experimental points in order to highlight characteristics shared by matrix/salt couples associated with low fluence values *vs* those leading to failed experiments (100% fluence). For that purpose, experimentally determined fluence threshold was added as an 11th variable to the set of the 10 descriptors (X_1_–X_10_). The PCA plots shown in Figure 1 reveal that the most discriminant variable accounting for failed MALDI of PS is the nature of the salt. On the one hand, the small angle formed by vectors associated to “fluence” and “cation radius” in Figure 1A reveals a positive correlation (r = +0.76) between these two variables. In other words, 100% fluence values are associated to experiments involving salt with high radius cation. PS adducts produced in the gas phase are usually described with the cation stacked between two adjacent phenyl groups (Gidden et al., 2002): our results suggest that such a conformation would be disfavored by large size cations. On the other hand, vectors associated to “salt bond energy” and “anion radius” form an angle close to 180° with the “fluence” vector (Figure 1A): these negative correlations (r = –0.50 and r = –0.64, respectively) indicate that 100% fluence values are associated to experiments involving salt with low radius anion or salt of low bond energy. These results are quite surprising since the amount of free cations available to interact with PS chains is expected to increase (and hence favor production of gas phase ions) as the bond energy of the salt decreases. Finally, with their vectors nearly orthogonal to the “fluence” vector in Figure 1A, any descriptors related to the matrix are poorly correlated with the fluence variable. Plotting the distribution of salts in this factorial plan (Figure 1B) clearly shows that rubidium and potassium salts all poorly perform (hence, in red) for MALDI of PS. A 100% fluence value is indeed assigned to all experiments conducted with KX or RbX salts (Supplementary Table 1), independently of the X counter-anion and of the matrix. Failure to acquire spectra of [PS + K]^+^ or [PS + Rb]^+^ was previously reported by Scrivens and coworkers when using dithranol as the matrix (Deery et al., 1997). In contrast, most data points associated to the use of silver or copper salts are located in the upper part of Figure 1B, indicating that these salts perform well for MALDI of PS. Of note, all detected ions in MALDI mass spectra were singly charged species, which means that Cu^II^ was reduced to Cu^I^ by electrons emitted from the metallic MALDI plate upon laser irradiation (Zhang et al., 2003). As detailed in Supplementary Table 1, fluence values below ∼40% were recorded for four of the six experiments involving Cu and three of the five experiments conducted with Ag. This result is consistent with the role of pre-formed matrix/salt clusters in the formation of PS ions in MALDI, as well as with the less pronounced clustering propensity of silver compared to copper, as demonstrated with dithranol (Lehmann et al., 1997). An intermediate situation is observed for lithium and sodium salts, with the success of MALDI experiments being matrix-dependent, as also reported by others (Deery et al., 1997). From these first results, it was decided to remove rubidium and potassium salts from the list of cationizing agents for PS. This led to withdrawal of 152 points from the candidate set (now composed of 342 points) and of 8 matrix/salt couples from the 25-point subset. The newly obtained subset of 17 points was badly conditioned and did not permit to estimate coefficients of a mathematical model. The uniform matrix was hence repaired by choosing new couples among the 342 remaining candidate points. Using the WSP algorithm and protecting the points already chosen, 16 new points were selected and experimentally tested: fluence values show failure of MALDI experiments in six cases, five of which involve the FA matrix (Supplementary Table 2).

**TABLE 3:** Experimental (^PS^Y_exp_) *vs* predicted (^PS^Y_calc_) fluence values when using the model built for PS with Ag and Cu metals (Eq. 1), with residuals (res) reported as ^PS^Y_exp_–^PS^Y_calc_.

Matrix/salt	^PS^Y_exp_	^PS^Y_calc_	Res
CMBT/Cu(NO_3_)_2_	22	21.5	+0.5
IAA/AgNO_3_	26	27.0	–1.0
FA/CuCl_2_	27	26.2	+0.8
FA/AgI	28	30.9	–2.9
5-CSA/AgI	29	28.6	+0.4
SA/Cu(NO_3_)_2_	31	32.0	–1.0
2,4-DHB/AgI	32	29.9	+2.1
HPA/AgNO_3_	40	40.6	–0.6
2,4-DHB/Cu(NO_3_)_2_	41	40.5	+0.5
NOR/CuCl	42	42.0	0
9-NA/AgI	44	43.4	+0.6
DHBQ/CuCl_2_	44	43.6	+0.4
HPA/CuCl_2_	45	46.5	–1.5
9-ACA/AgNO_3_	45	43.4	+1.6

**FIGURE 2:**
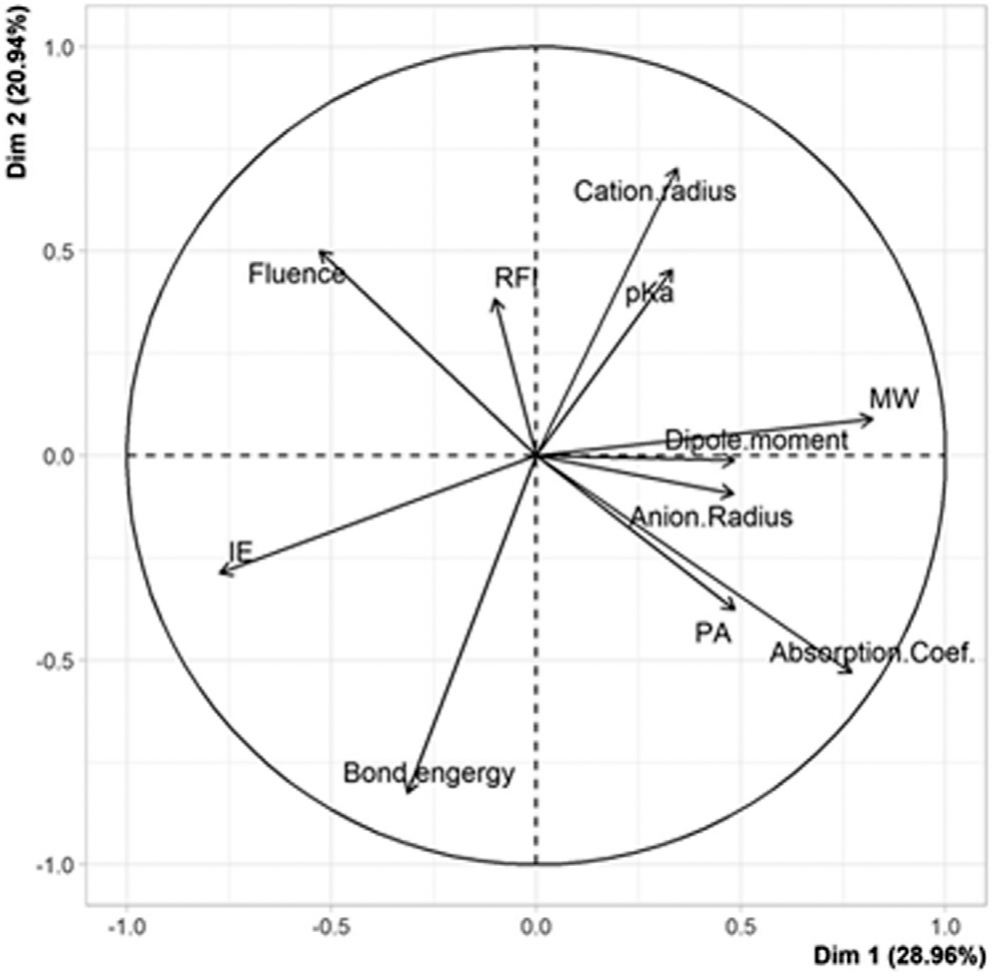
PCA plot of variables for the 14 couples involving Ag or Cu salts tested for MALDI of PS.

The model built when considering *N* = 33 points (17 remaining couples in Supplementary Table 1 + the 16 couples of Supplementary Table 2) was quite bad at predicting correct fluence values and the behavior of the different couples remained very difficult to understand (data not shown). It was thus decided to split the learning set based on the nature of the cation, that is, metals such as Ag and Cu that systematically allow successful MALDI of PS *vs* alkali (Na and Li) with which production of cationized PS is matrix-dependent. The PCA plot obtained for the group of 14 couples involving either Ag or Cu salt is shown in Figure 2. A single positive correlation is observed between the laser fluence and matrix fluorescence: this matrix property is well-known to be detrimental to the MALDI process but the measured correlation remains quite low (r = +0.28). In contrast, a strong negative correlation (r = –0.75) is clearly revealed in Figure 2 between the laser fluence and the absorption coefficient of the matrix (ε_337_). Another negative correlation, yet less pronounced (r = –0.28), is found between laser fluence and proton affinity, used here to illustrate cation affinity of the matrix. This last result supports the current opinion about PS cationization occurring *via* a gas phase process (Lehmann et al., 1997): when using matrices with high cation affinity, energy input has to be increased for cations to be transferred from the matrix/salt clusters to PS chains.

The model constructed with a stepwise regression based on the subset of 14 points available for Ag and Cu salts is defined by Eq. 1. As reported in Table 3, this model allows excellent predictions of the fluence, with *R* ^2^ = 0.976 and residuals below ±3.

$$\begin{array}{l} Y=46.89-1.67X_{1}+5.50X_{2}-1.452X_{6}+10.75X_{8}-6.10X_{9}+11.44X_{10}+18.45X_{8}X_{10}+4.98X_{1}X_{8}-26.25X_{2}X_{9} \end{array}$$ (1)

As compared to this first model, the second model constructed for PS with the subset of 19 points related to alkali salts is slightly less efficient at predicting accurate fluence (*R* ^2^ = 0.874). It is defined by Eq. 2 and yields ^PS^Y_calc_ data with absolute value of residuals ranging from 0.5 to 22.3 (Table 4).

$$\begin{array}{l} Y=54.91+45.31X_{1}-19.00X_{4}-2.87X_{6}-23.25X_{7}+3.98X_{8}+2.56X_{10}-108.89X_{4}X_{6}+53.48X_{6}X_{10}+18.86X_{7}X_{8} \end{array}$$ (2)

**TABLE 4:** Experimental (^PS^Y_exp_) *vs* predicted (^PS^Y_calc_) fluence values when using the model built for PS with alkali salts (Eq. 2), with residuals (res) reported as ^PS^Y_exp_–^PS^Y_calc_.

Matrix/salt	^PS^Y_exp_	_PS_Ycalc	Res
HCCA/LiI	25	37.6	–12.6
FA/LiF	31	53.3	–22.3
CMBT/LiF	32	24.1	+7.9
2,6-DHB/NaF	34	28.7	+5.3
HABA/LiBr	42	48.8	–6.8
THAP/NaBr	43	37.9	+5.1
9-NA/LiF	46	48.5	–2.5
2,4-DHB/NaI	58	60.2	–2.2
2,4-DHB/LiI	60	62.4	–2.4
DHBQ/NaI	60	51.9	+8.1
2,4-DHB/NaCl	63	71.4	–8.4
2,4-DHB/LiF	100	107.0	–7.0
FA/LiCl	100	78.9	+21.1
FA/LiBr	100	85.8	+14.2
FA/LiI	100	95.0	+5.0
FA/NaCl	100	100.5	–0.5
FA/NaI	100	115.0	–15.0
HABA/NaF	100	92.4	+7.6
HPA/LiF	100	94.7	+5.3

However, nearly 74% of these predictions were achieved with a residual value below ±10 and, most importantly, the model allows unsuccessful experiments (fluence 100%) to be clearly discriminated from those yielding PS ions upon MALDI. With both models in hands, predicted fluence values were calculated for the 494 matrix/salt combinations (Supplementary Tables 4–13) and will be considered together with predictions obtained for PEG to rationalize the selection of matrix/salt couples for MALDI of PEO-*b*-PS block copolymer (*vide infra*).

### QSAR Study of PEG

The same 25 couples as first used in the case of PS were considered in the QSAR study of PEG. However, results of MALDI experiments led us to remove some couples from this subset. The CMBT/LiF couple was no longer considered because it led to mass data strongly lacking reproducibility. Three additional couples were removed to avoid biased results due to spectral interferences between the targeted species and ionic adducts formed with residual sodium of polluted matrices. For example, safe distinction could not be achieved between [PEG_n–1_ + Cu]^+^ and [PEG_n_ + Na]^+^ ions in MALDI experiments conducted with 2,4–DBH/Cu(NO_3_)_2_ and CMBT/Cu(NO_3_)_2_. Similarly [PEG_n–2_ + Ag]^+^ could not be resolved from [PEG_n_ + Na]^+^ when using the 9-ACA/AgNO_3_ couple. Using the WSP algorithm, addition of three new matrix/salt pairs (namely, MBT/AgNO_3_, HPA/Cu(NO_3_)_2_ and HCCA/CuCl_2_) was found to be sufficient to repair the matrix.

**FIGURE 3:**
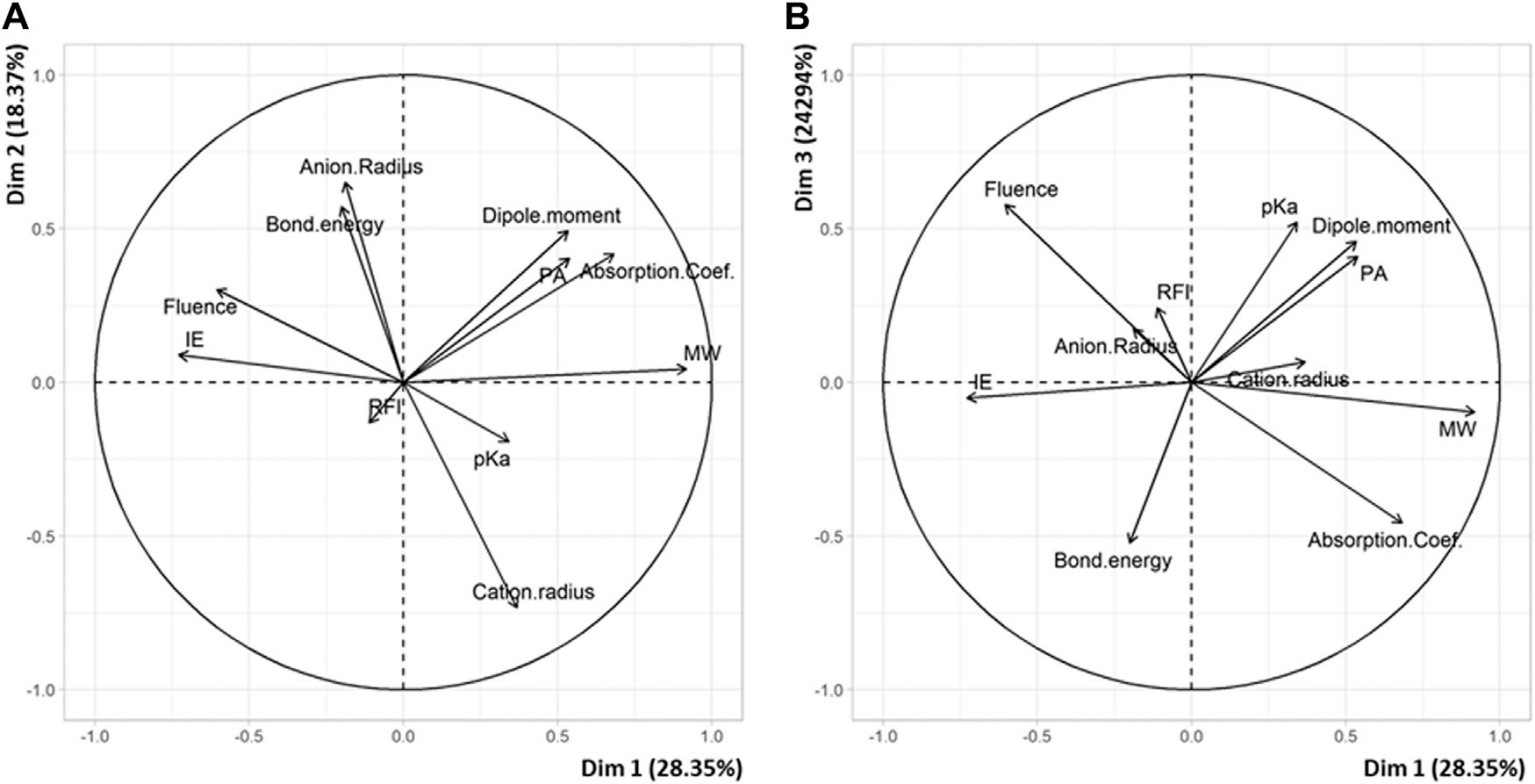
PCA plots of variables in **(A)** dimension 2 *vs* dimension 1 and **(B)** dimension 3 *vs* dimension 1 for the 24 matrix/salt couples tested for MALDI of PEG.

The PCA plot built with data obtained for PEG with the new 24-point subset (Supplementary Table 3) allows three main matrix descriptors to be identified for their influence on laser fluence (Figure 3). First, the positive correlation (r = +0.29) observed between laser fluence and the ionization energy of the matrix indicates that the highest fluence values are most often found for those matrix having high ionization energy (Figure 3A). This result is consistent with the coupled chemical and physical dynamics model proposed by Knochenmuss (Knochenmuss, 2003): formation of matrix ions is the key primary event in UV-MALDI since analyte ions observed in mass spectra are predominantly formed *via* secondary ion-molecule reactions with these matrix ions in the MALDI plume (Knochenmuss and Zenobi, 2003). Two other matrix descriptors exhibit negative correlation with the laser fluence when plotting data in a second set of dimensions (Figure 3B). Matrices with low ε_337_ absorption coefficient require high fluence to induce the MALDI process (r = –0.47), which is a quite obvious relationship. In contrast, the reason why matrices of low molecular weight are often found in matrix/salt associated with high fluence (r = –0.53) is more puzzling. Yet, using solid state NMR, our group has recently shown that, in the case of 2,x-DHB isomers (with x = 3–6), MALDI of PEG was influenced by matrix/polymer aggregates formed in the solid state upon grinding (Pizzala et al., 2021). A similar study should be conducted to find out whether such aggregates are also formed with alternative matrices and, if so, whether the number of molecules in such aggregates varies with the size, and hence the molecular weight, of the matrix. The PCA plot of Figure 3A also highlights the influence of the salt properties. The negative correlation between laser fluence and the cation radius (r = –0.43) indicates that low fluence is required to desorb PEG adducted with large cation: this is consistent with the quite long PEG chains studied here (*M*_n_ = 4 kDa) requiring rather big cation to form stable adducts in the gas phase (Jackson et al., 1997). A positive correlation is also noted between laser fluence and the anion radius (r = +0.36), although it remains hard to interpret, at least in terms of salt bond energy since the latter parameter was found to be of negligible influence (r = +0.05). Overall, unlike for PS, distribution of matrix couples in this factorial plane does not exhibit any cleavage between individuals (Supplementary Figure 1). Accordingly, the predictive model for PEG defined by Eq. 3 was built using all data obtained for these 24 matrix/salt couples.

$$\begin{array}{l} Y=57.89-23.99X_{1}-33.30X_{2}+15.63X_{3}-4.25X_{4}-13.95X_{5}-15.565X_{6}-30.69X_{8}-1.23X_{10}+29.42X_{1}X_{2}-67.97X_{2}X_{8}+24.715X_{1}X_{8}+9.38X_{3}X_{5} \end{array}$$ (3)

With *R* ^2^ = 0.78, this PEG model is less accurate than the two models built for PS, although 75% of predicted values have a residual value below ±15 (Table 5). Moreover, discrimination of unsuccessful experiments (fluence 100%) is not achieved in a few cases. For example, the fluence threshold of ∼75% predicted for failed experiments conducted with AgNO_3_ and either IAA or MBT is lower than the 83% value predicted for the THAP/NaBr couple experimentally found to produce MALDI data from 51% fluence threshold. In spite of these few limitations, this model was applied to predict fluence values in MALDI of PEG for the 494 matrix/salt combinations (Supplementary Tables 4–13).

**TABLE 5:** Experimental (^PEG^Y_exp_) *vs* predicted (^PEG^Y_calc_) fluence values when using the model built for PEO, with residuals (res) reported as ^PEG^Y_exp_–^PEG^Y_calc_.

Matrix/salt	^PEG^Y_exp_	^PEG^Y_calc_	Res
HABA/LiBr	24	12.7	+11.3
HCCA/CuCl_2_	27	35.2	–8.2
CMBT/RbF	29	32.6	–3.6
5-CSA/AgI	31	29.6	+1.4
SA/Cu(NO_3_)_2_	33	31.4	+1.6
SA/KI	33	47.9	–14.9
9-NA/RbF	35	34.8	+0.2
HABA/KF	35	42.4	–7.4
FA/CuCl_2_	39	51.8	–12.8
2,5-DHB/KF	45	57.8	–12.8
9-ACA/RbCl	48	33.4	+14.6
DHBQ/NaI	48	69.1	–21.1
HPA/RbCl	49	49.9	–0.9
THAP/NaBr	51	83.9	–32.9
2,4-DHB/RbCl	71	58.8	+12.2
2,4-DHB/LiF	73	84.2	–11.2
9-NA/AgI	100	99.3	+0.7
DHBQ/CuCl_2_	100	84.6	+15.4
HPA/AgNO_3_	100	82.6	+17.4
HPA/Cu(NO_3_)_2_	100	99.6	+0.4
HPA/LiF	100	97.4	+2.6
IAA/AgNO_3_	100	74.0	+26.0
MBT/AgNO_3_	100	75.4	+24.6
NOR/CuCl	100	103.0	–3.0

### MALDI of the PEO_1800_-*b*-PS_1600_ Copolymer

**FIGURE 4:**
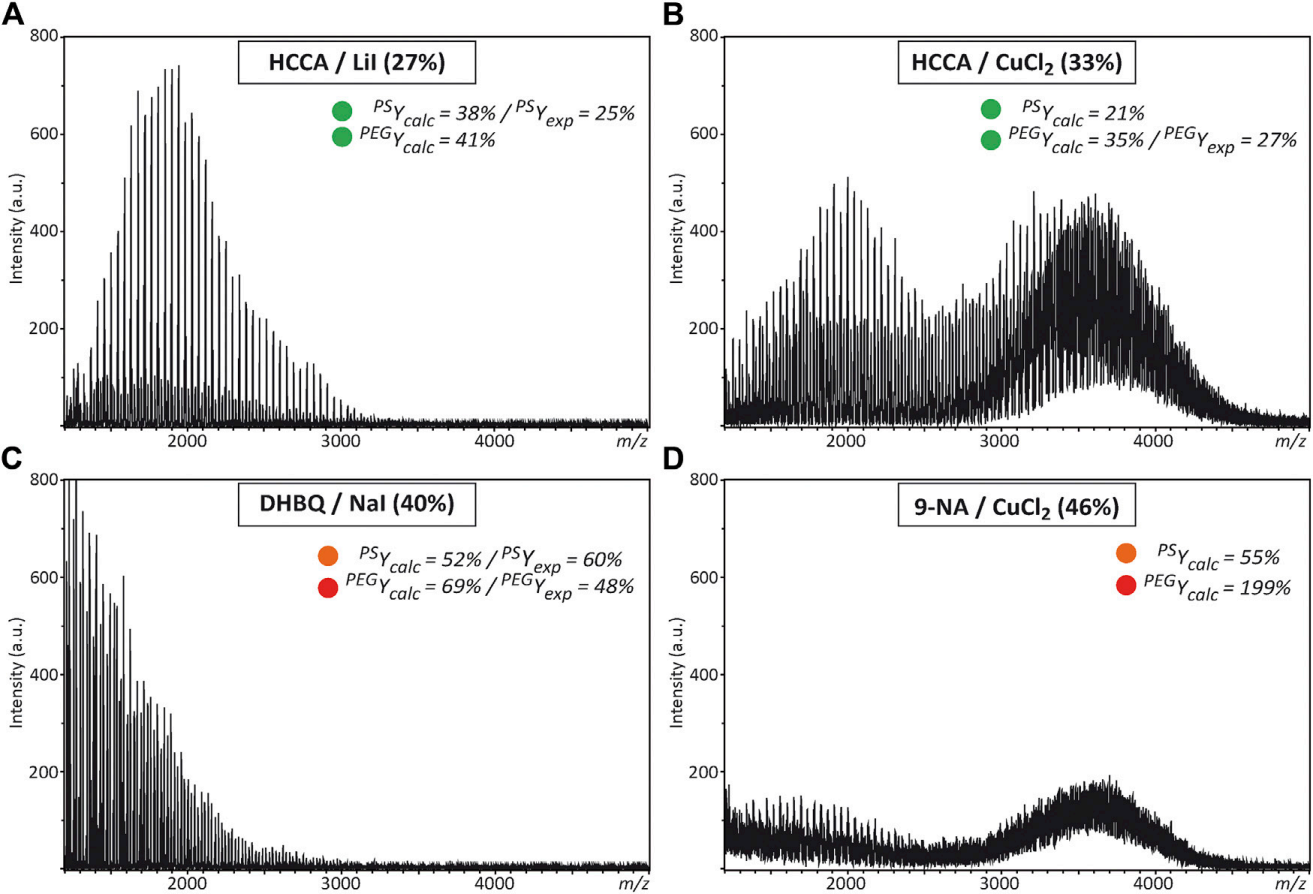
MALDI mass spectra recorded for the PEO-*b*-PS copolymer when using **(A)** HCCA/LiI **(B)** HCCA/CuCl_2_ **(C)** DHBQ/NaI, or (D) 9-NA/CuCl_2_ as the matrix/salt couple, with the employed laser fluence into parenthesis. Inset: Predicted (Y_calc_) or experimental (Y_exp_) laser fluence when these experimental conditions are employed for PS and PEG homopolymers, using the same color code as in Supplementary Figures 4-13 to qualify laser fluence requirement (≤50%, in green; 51–75%, in orange; > 75%, in red).

As previously mentioned, the search for appropriate experimental conditions enabling successful MALDI of the targeted block copolymer relies here on the assumption that any matrix/salt couple found to properly operate (*i.e.*, with low fluence threshold) for MALDI of both PEG and PS homopolymers would be relevant candidates to be tested for MALDI of PEO-*b*-PS copolymers. Accordingly, predicted ^PEG^Y_calc_ and ^PS^Y_calc_ values listed in Supplementary Tables 4–13 were compared for all matrix couples, using a color code to rapidly identify which conditions were associated to low (≤50%, in green), medium (51–75%, in orange) or high (>75%, in red) laser fluence requirement. Then, selected matrix/salt couples were tested for the PEO_1800_-*b*-PS_1600_ copolymer. Detailed analysis of these results is beyond the scope of the present study, so only typical examples are shown in Figure 4 and Supplementary Figure 2. On the one hand, experimental conditions shown to work for both homopolymers often failed at producing MALDI data for the copolymer. For example, data obtained with HCCA/LiI (Figure 4A) revealed the presence of free PEO in the studied sample (which was confirmed by liquid chromatography analysis reported in Supplementary Figure 3) but no trace of the copolymeric distribution. Even worse results were obtained with the 5-CSA/AgI couple that mainly generates [AgI]_n_Ag^+^ salt clusters in MALDI (Supplementary Figure 2A). This particular result shows that the analyte, alone or in conjunction with the matrix, actually contribute to the extent of salt dissociation upon grinding, as previously demonstrated in the case of PEG with DHB matrices (Pizzala et al., 2021). Accordingly, one way to further improve the DoE performance should be to include specific properties of the copolymer as additional variables. In contrast, employing HCCA/CuCl_2_ permits to generate the targeted copolymeric distribution centered at the expected *m/z* ∼ 3,600 (Figure 4B), consistent with the efficiency of this couple to promote MALDI of individual homopolymers. Detailed assignment of signals measured for co-oligomers is provided in Supplementary Figure 4. On the other hand, MALDI of the studied PEO-*b*-PS copolymer could also be achieved with some matrix/salt systems that were shown to badly perform for constituting segments, as depicted in Figure 4D with 9-NA/CuCl_2_. Yet, again, this is not a systematic rule, as exemplified in Figure 4C with the MALDI mass spectrum obtained with DHBQ/NaI mainly showing signals of residual PEO. Overall, these results show that chains composed of PEO and PS segments do not behave as a combination of the two polymeric species in MALDI, hence ruling out our initial assumption. Alternatively, proper modelling of the MALDI behavior of synthetic polymers would require not only matrix and salt parameters to be considered but also properties of the analyte itself. In other words, any DoE aimed at optimizing MALDI experimental conditions of amphiphilic block copolymers should be developed for the targeted copolymer itself rather than its constituting homopolymers. The approach developed here has permitted to identify some matrix/salt couples that promote ionization of the PEO-*b*-PS block copolymer, which means that the set of 19 matrices and 26 salts used in the present study can be safely used to perform a new DoE based on QSAR analysis for MALDI of PEO-*b*-PS.

## Conclusion

In this study, 19 matrices and 26 salts were considered for a design of experiments aimed at studying the influence of 10 variables (7 for each matrix, 3 for each salt) on MALDI data of PEG and PS homopolymers, in order to further build models that can predict best experimental conditions, as measured by the lowest laser fluence threshold requested to achieve ionization. To do so, WSP algorithm was used to define reduced subsets based the uniformity of their distribution in the space of the 10 descriptors. For PS, the subset had to be split into two groups, containing 14 and 19 couples, respectively, while a single subset of 24 couples was tested for PEG. Predictive models built with QSAR were found to be quite reliable in spite of the simplicity of the singly monitored response (*i.e.*, laser fluence threshold) which enabled fast measurement while avoiding extensive mass data analysis. It should however be acknowledged that the time-limiting step was the solvent-free sample preparation, but this method was observed here to produce quite homogeneous solid mixtures while suppressing any additional (influential) factors introduced by the use of solvents. Although this was not the main goal here, this study also permitted to highlight some general correlations between individual properties of matrix and salt and the MALDI efficiency of matrix/salt combinations towards PEG and PS. For example, it was found that the nature of the salt is the key factor in MALDI of PS whereas successful MALDI of PEG is also matrix-dependent. This makes this DoE approach highly valuable in fundamental works conducted to best understand MALDI. However, some limitations were also identified, particularly in the predictive model built for PEG, which suggests that some influential parameters were not taken into account. Most importantly, this study showed that, in terms of MALDI behavior, a block copolymer of PS and PEO is not exactly a combination of these two species, which means that DoE-based optimization of such an amphiphilic copolymer should be developed using the copolymer itself rather than its constituting blocks as models.

## Data Availability

The raw data supporting the conclusion of this article will be made available by the authors, without undue reservation.
