# Supplementary material for: Design of Experiments for Matrix-Assisted Laser Desorption/Ionization of Amphiphilic Poly(Ethylene Oxide)-b-Polystyrene Block Copolymers
Source: Front Chem. 2021 Sep 9;9:740495. doi: 10.3389/fchem.2021.740495 (PMC8458736; doi:10.3389/fchem.2021.740495)
Supplement: Supplementary file 1 [file DataSheet1.PDF]

Supplementary Material

Content	page
1. QSAR of PS	
Supplementary Table 1. $^{PS}Y_{exp}$ when testing the 25 matrix/salt initial combinations ……….	S2
Supplementary Table 2. $^{PS}Y_{exp}$ when testing the 16 matrix/salt additional combinations …..	S3
2. QSAR of PEG	
Supplementary Table 3. $^{PEG}Y_{exp}$ when testing the 24 matrix/salt combinations …………….	S4
Supplementary Figure 1. PCA plots of individuals for MALDI of PEG …………………….	S5
3. Predicted laser fluence threshold for PS and PEG	
Suppl. Table 4. $^{PS}Y_{calc}$ and $^{PEG}Y_{calc}$ with 2,3-DHB and 2,4-DHB ………………………….	S6
Suppl. Table 5. $^{PS}Y_{calc}$ and $^{PEG}Y_{calc}$ with 2,5-DHB and 2,6-DHB ………………………….	S7
Suppl. Table 6. $^{PS}Y_{calc}$ and $^{PEG}Y_{calc}$ with 5-CSA and 9-ACA ………………………………..	S8
Suppl. Table 7. $^{PS}Y_{calc}$ and $^{PEG}Y_{calc}$ with 9-NA and CMBT …………………………………	S9
Suppl. Table 8. $^{PS}Y_{calc}$ and $^{PEG}Y_{calc}$ with DHBQ and Dithranol ……………………………..	S10
Suppl. Table 9. $^{PS}Y_{calc}$ and $^{PEG}Y_{calc}$ with FA and HABA ……………………………………	S11
Suppl. Table 10. $^{PS}Y_{calc}$ and $^{PEG}Y_{calc}$ with HCCA and HPA …………………………………	S12
Suppl. Table 11. $^{PS}Y_{calc}$ and $^{PEG}Y_{calc}$ with IAA and MBT …………………………………...	S13
Suppl. Table 12. $^{PS}Y_{calc}$ and $^{PEG}Y_{calc}$ with NOR and SA …………………………………….	S14
Suppl. Table 13. $^{PS}Y_{calc}$ and $^{PEG}Y_{calc}$ with THAP …………………………………………...	S15
4. Analysis of the PEO-*b*-PS block copolymer	
Suppl. Figure 2. MALDI of PEO-*b*-PS: 5-CSA/AgI, HABA/LiBr, THAP/NaBr, or SA/NaF	S16
Suppl. Figure 3. LC-LCD of the PEO-*b*-PS copolymer ……….……………………………	S17
Suppl. Figure 4. Peak assignments in MALDI-MS of PEO-*b*-PS …………………………..	S18
5. Complete list of authors for Gaussian software …………………………..………………	S19

1 QSAR of PS

Supplementary Table 1. Laser fluence threshold determined for MALDI of PS ($^{PS}Y_{exp}$) when testing the 25 matrix/salt combinations.

matrix	salt	$^{PS}Y_{exp}$ (%)
CMBT	$Cu(NO_3)_2$	22
IAA	$AgNO_3$	26
FA	$CuCl_2$	27
5-CSA	AgI	29
SA	$Cu(NO_3)_2$	31
CMBT	LiF	32
HPA	$AgNO_3$	40
2,4-DHB	$Cu(NO_3)_2$	41
HABA	LiBr	42
NOR	CuCl	42
THAP	NaBr	43
9-NA	AgI	44
DHBQ	$CuCl_2$	44
9-ACA	$AgNO_3$	45
DHBQ	NaI	60
2,4-DHB	LiF	100
HPA	LiF	100
2,5-DHB	KF	100
HABA	KF	100
SA	KI	100
2,4-DHB	RbCl	100
9-ACA	RbCl	100
9-NA	RbF	100
HPA	RbCl	100
CMBT	RbF	100

Supplementary Table 2. Laser fluence threshold determined for MALDI of PS ($^{PS}Y_{exp}$) when testing the 16 additional matrix/salt combinations.

matrix	salt	$^{PS}Y_{exp}$ (%)
HCCA	LiI	25
FA	AgI	28
FA	LiF	31
2,4-DHB	AgI	32
2,6-DHB	NaF	34
HPA	$CuCl_2$	45
9-NA	LiF	46
2,4-DHB	NaI	58
2,4-DHB	LiI	60
2,4-DHB	NaCl	63
FA	LiCl	100
FA	LiBr	100
FA	LiI	100
FA	NaI	100
FA	NaCl	100
HABA	NaF	100

2 Supplementary Data for QSAR of PEG

Supplementary Table 3. Laser fluence threshold measured for MALDI of PEG ($^{PEG}Y_{exp}$) when testing the 24 matrix/salt combinations.

	matrix	salt	$^{PEG}Y_{exp}$ (%)
1	HABA	LiBr	24
2	HCCA	$CuCl_2$	27
3	CMBT	RbF	29
4	5-CSA	AgI	31
5	SA	$Cu(NO_3)_2$	33
6	SA	KI	33
7	9-NA	RbF	35
8	HABA	KF	35
9	FA	$CuCl_2$	39
10	2,5-DHB	KF	45
11	9-ACA	RbCl	48
12	DHBQ	NaI	48
13	HPA	RbCl	49
14	THAP	NaBr	51
15	2,4-DHB	$Cu(NO_3)_2$	63
16	2,4-DHB	RbCl	71
17	2,4-DHB	LiF	73
18	9-ACA	$AgNO_3$	100
19	9-NA	AgI	100
20	CMBT	$Cu(NO_3)_2$	100
21	CMBT	LiF	100
22	DHBQ	$CuCl_2$	100
23	HPA	$AgNO_3$	100
24	HPA	$Cu(NO_3)_2$	100
25	HPA	LiF	100
26	IAA	$AgNO_3$	100
27	MBT	$AgNO_3$	100
28	NOR	CuCl	100

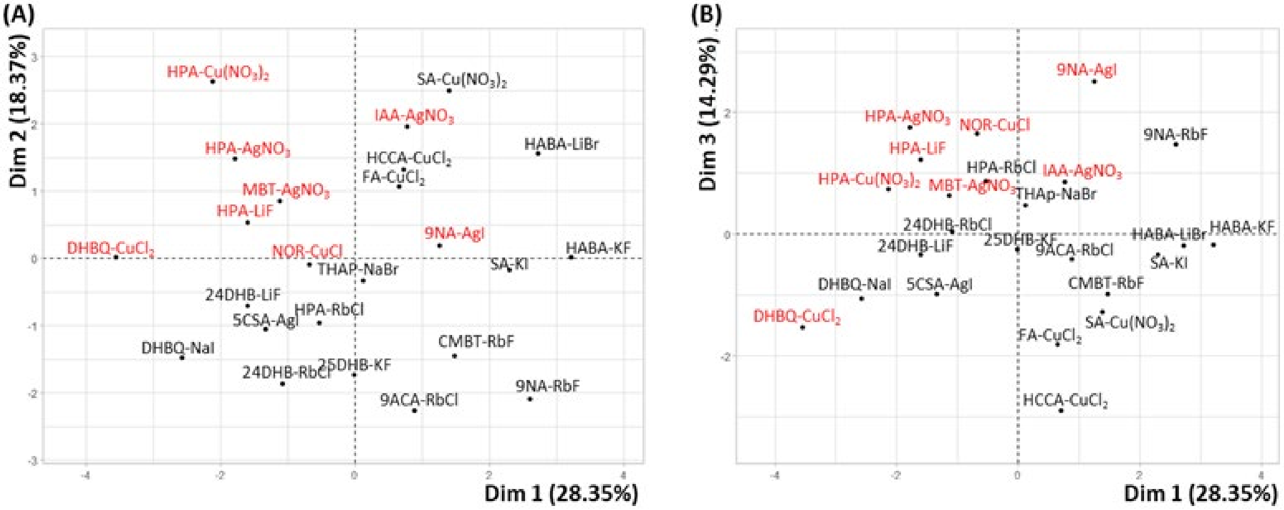

Supplementary Figure 1. PCA plots of individuals in (A) dimension 2 *vs* dimension 1 and (B) dimension 3 *vs* dimension 1 for the 24 matrix/salt couples tested for MALDI of PEG.

3 Predicted laser fluence threshold for PS and PEG

Supplementary Table 4. Laser fluence threshold predicted for PS (${}^{PS}Y_{calc}$, in %) and for PEG (${}^{PEG}Y_{calc}$, in %) for the 26 matrix/salt couples involving 2,3-DHB (left) or 2,4-DHB (right). The second value into parentheses was determined experimentally. Color code: $Y_{calc} \leq 50\%$, green; $50\% < Y_{calc} \leq 75\%$, orange; $75\% < Y_{calc}$, red. Empty grey cells designate undetermined data (see text).

matrix	salt	${}^{PS}Y_{calc}$	${}^{PEG}Y_{calc}$
2,3-DHB	AgF	22	69
2,3-DHB	AgCl	23	69
2,3-DHB	AgBr	25	69
2,3-DHB	AgI	27	69
2,3-DHB	$AgNO_3$	30	73
2,3-DHB	CuCl	39	83
2,3-DHB	CuBr	41	83
2,3-DHB	CuI	44	83
2,3-DHB	$CuCl_2$	27	82
2,3-DHB	$Cu(NO_3)_2$	39	78
2,3-DHB	LiF	35	79
2,3-DHB	LiCl	32	79
2,3-DHB	LiBr	31	79
2,3-DHB	LiI	30	79
2,3-DHB	NaF	32	72
2,3-DHB	NaCl	30	72
2,3-DHB	NaBr	29	73
2,3-DHB	NaI	28	73
2,3-DHB	KF		63
2,3-DHB	KCl		64
2,3-DHB	KBr		64
2,3-DHB	KI		64
2,3-DHB	RbF		60
2,3-DHB	RbCl		60
2,3-DHB	RbBr		60
2,3-DHB	RbI		60

matrix	salt	${}^{PS}Y_{calc}$	${}^{PEG}Y_{calc}$
2,4-DHB	AgF	27	71
2,4-DHB	AgCl	28	71
2,4-DHB	AgBr	29	71
2,4-DHB	AgI	30 (32)	71
2,4-DHB	$AgNO_3$	32	76
2,4-DHB	CuCl	43	90
2,4-DHB	CuBr	45	90
2,4-DHB	CuI	47	90
2,4-DHB	$CuCl_2$	31	89
2,4-DHB	$Cu(NO_3)_2$	40 (41)	
2,4-DHB	LiF	107 (100)	84 (73)
2,4-DHB	LiCl	75	85
2,4-DHB	LiBr	70	85
2,4-DHB	LiI	62 (60)	85
2,4-DHB	NaF	87	75
2,4-DHB	NaCl	71 (63)	76
2,4-DHB	NaBr	67	76
2,4-DHB	NaI	60 (58)	76
2,4-DHB	KF		63
2,4-DHB	KCl		64
2,4-DHB	KBr		64
2,4-DHB	KI		64
2,4-DHB	RbF		59
2,4-DHB	RbCl		59 (71)
2,4-DHB	RbBr		59
2,4-DHB	RbI		59

Supplementary Table 5. Laser fluence threshold predicted for PS (${}^{PS}Y_{calc}$, in %) and for PEG (${}^{PEG}Y_{calc}$, in %) for the 26 matrix/salt couples involving 2,5-DHB (left) or 2,6-DHB (right). The second value into parentheses was determined experimentally. Color code: $Y_{calc} \leq 50\%$, green; $50\% < Y_{calc} \leq 75\%$, orange; $75\% < Y_{calc}$, red. Empty grey cells designate undetermined data (see text).

matrix	salt	${}^{PS}Y_{calc}$	${}^{PEG}Y_{calc}$	matrix	salt	${}^{PS}Y_{calc}$	${}^{PEG}Y_{calc}$
2,5-DHB	AgF	20	63	2,6-DHB	AgF	6	72
2,5-DHB	AgCl	21	64	2,6-DHB	AgCl	14	72
2,5-DHB	AgBr	23	64	2,6-DHB	AgBr	20	72
2,5-DHB	AgI	25	64	2,6-DHB	AgI	25	72
2,5-DHB	$AgNO_3$	28	68	2,6-DHB	$AgNO_3$	39	66
2,5-DHB	CuCl	37	78	2,6-DHB	CuCl	29	49
2,5-DHB	CuBr	39	78	2,6-DHB	CuBr	36	49
2,5-DHB	CuI	42	78	2,6-DHB	CuI	42	49
2,5-DHB	$CuCl_2$	25	77	2,6-DHB	$CuCl_2$	17	46
2,5-DHB	$Cu(NO_3)_2$	36	73	2,6-DHB	$Cu(NO_3)_2$	47	52
2,5-DHB	LiF	20	73	2,6-DHB	LiF	23	56
2,5-DHB	LiCl	28	74	2,6-DHB	LiCl	25	56
2,5-DHB	LiBr	30	74	2,6-DHB	LiBr	25	56
2,5-DHB	LiI	33	74	2,6-DHB	LiI	26	56
2,5-DHB	NaF	26	67	2,6-DHB	NaF	29 (34)	67
2,5-DHB	NaCl	32	67	2,6-DHB	NaCl	30	67
2,5-DHB	NaBr	34	67	2,6-DHB	NaBr	30	67
2,5-DHB	NaI	36	67	2,6-DHB	NaI	31	67
2,5-DHB	KF		58 (45)	2,6-DHB	KF		82
2,5-DHB	KCl		58	2,6-DHB	KCl		82
2,5-DHB	KBr		58	2,6-DHB	KBr		82
2,5-DHB	KI		58	2,6-DHB	KI		82
2,5-DHB	RbF		54	2,6-DHB	RbF		88
2,5-DHB	RbCl		54	2,6-DHB	RbCl		88
2,5-DHB	RbBr		54	2,6-DHB	RbBr		88
2,5-DHB	RbI		55	2,6-DHB	RbI		88

Supplementary Table 6. Laser fluence threshold predicted for PS ($^{PS}Y_{calc}$, in %) and for PEG ($^{PEG}Y_{calc}$, in %) for the 26 matrix/salt couples involving 5-CSA (left) or 9-ACA (right). The second value into parentheses was determined experimentally. Color code: $Y_{calc} \leq 50\%$, green; $50\% < Y_{calc} \leq 75\%$, orange; $75\% < Y_{calc}$, red. Empty grey cells designate undetermined data (see text).

matrix	salt	$^{PS}Y_{calc}$	$^{PEG}Y_{calc}$	matrix	salt	$^{PS}Y_{calc}$	$^{PEG}Y_{calc}$
5-CSA	AgF	22	29	9-ACA	AgF	41	23
5-CSA	AgCl	24	30	9-ACA	AgCl	42	23
5-CSA	AgBr	27	30	9-ACA	AgBr	42	23
5-CSA	AgI	29 (29)	30 (31)	9-ACA	AgI	43	23
5-CSA	$AgNO_3$	34	29	9-ACA	$AgNO_3$	43 (45)	
5-CSA	CuCl	36	27	9-ACA	CuCl	44	9
5-CSA	CuBr	39	27	9-ACA	CuBr	45	9
5-CSA	CuI	42	27	9-ACA	CuI	47	9
5-CSA	$CuCl_2$	24	26	9-ACA	$CuCl_2$	32	6
5-CSA	$Cu(NO_3)_2$	39	26	9-ACA	$Cu(NO_3)_2$	39	10
5-CSA	LiF	98	28	9-ACA	LiF	67	13
5-CSA	LiCl	83	28	9-ACA	LiCl	119	13
5-CSA	LiBr	79	28	9-ACA	LiBr	133	13
5-CSA	LiI	73	28	9-ACA	LiI	152	14
5-CSA	NaF	64	29	9-ACA	NaF	81	20
5-CSA	NaCl	52	29	9-ACA	NaCl	123	20
5-CSA	NaBr	48	29	9-ACA	NaBr	135	20
5-CSA	NaI	43	30	9-ACA	NaI	153	20
5-CSA	KF		31	9-ACA	KF		29
5-CSA	KCl		31	9-ACA	KCl		30
5-CSA	KBr		31	9-ACA	KBr		30
5-CSA	KI		31	9-ACA	KI		30
5-CSA	RbF		31	9-ACA	RbF		33
5-CSA	RbCl		31	9-ACA	RbCl		33 (48)
5-CSA	RbBr		31	9-ACA	RbBr		33
5-CSA	RbI		31	9-ACA	RbI		34

Supplementary Table 7. Laser fluence threshold predicted for PS (${}^{PS}Y_{calc}$, in %) and for PEG (${}^{PEG}Y_{calc}$, in %) for the 26 matrix/salt couples involving 9-NA (left) or CMBT (right). The second value into parentheses was determined experimentally. Color code: $Y_{calc} \leq 50\%$, green; $50\% < Y_{calc} \leq 75\%$, orange; $75\% < Y_{calc}$, red. Empty grey cells designate undetermined data (see text).

matrix	salt	${}^{PS}Y_{calc}$	${}^{PEG}Y_{calc}$
9-NA	AgF	84	99
9-NA	AgCl	65	99
9-NA	AgBr	54	99
9-NA	AgI	43 (44)	99 (100)
9-NA	$AgNO_3$	12	126
9-NA	CuCl	67	195
9-NA	CuBr	56	195
9-NA	CuI	48	195
9-NA	$CuCl_2$	55	199
9-NA	$Cu(NO_3)_2$	7	176
9-NA	LiF	49 (46)	167
9-NA	LiCl	87	168
9-NA	LiBr	97	168
9-NA	LiI	111	168
9-NA	NaF	81	122
9-NA	NaCl	112	122
9-NA	NaBr	121	122
9-NA	NaI	134	132
9-NA	KF		59
9-NA	KCl		59
9-NA	KBr		59
9-NA	KI		60
9-NA	RbF		35 (35)
9-NA	RbCl		35
9-NA	RbBr		35
9-NA	RbI		35

matrix	salt	${}^{PS}Y_{calc}$	${}^{PEG}Y_{calc}$
CMBT	AgF	39	49
CMBT	AgCl	34	49
CMBT	AgBr	32	49
CMBT	AgI	30	49
CMBT	$AgNO_3$	22	56
CMBT	CuCl	40	74
CMBT	CuBr	39	74
CMBT	CuI	38	74
CMBT	$CuCl_2$	28	74
CMBT	$Cu(NO_3)_2$	21 (22)	
CMBT	LiF	24 (32)	
CMBT	LiCl	10	67
CMBT	LiBr	7	67
CMBT	LiI	2	67
CMBT	NaF	39	55
CMBT	NaCl	28	55
CMBT	NaBr	25	56
CMBT	NaI	21	56
CMBT	KF		39
CMBT	KCl		39
CMBT	KBr		39
CMBT	KI		39
CMBT	RbF		33 (29)
CMBT	RbCl		33
CMBT	RbBr		33
CMBT	RbI		33

Supplementary Table 8. Laser fluence threshold predicted for PS ($^{PS}Y_{calc}$, in %) and for PEG ($^{PEG}Y_{calc}$, in %) for the 26 matrix/salt couples involving DHBQ (left) or Dithranol (right). The second value into parentheses was determined experimentally. Color code: $Y_{calc} \leq 50\%$, green; $50\% < Y_{calc} \leq 75\%$, orange; $75\% < Y_{calc}$, red. Empty grey cells designate undetermined data (see text).

matrix	salt	$^{PS}Y_{calc}$	$^{PEG}Y_{calc}$
DHBQ	AgF	36	63
DHBQ	AgCl	37	63
DHBQ	AgBr	39	63
DHBQ	AgI	40	64
DHBQ	$AgNO_3$	44	70
DHBQ	CuCl	55	85
DHBQ	CuBr	58	85
DHBQ	CuI	60	85
DHBQ	$CuCl_2$	44 (44)	85 (100)
DHBQ	$Cu(NO_3)_2$	55	79
DHBQ	LiF	194	79
DHBQ	LiCl	136	79
DHBQ	LiBr	121	79
DHBQ	LiI	100	79
DHBQ	NaF	130	69
DHBQ	NaCl	85	69
DHBQ	NaBr	71	69
DHBQ	NaI	52 (60)	69 (48)
DHBQ	KF		55
DHBQ	KCl		55
DHBQ	KBr		55
DHBQ	KI		55
DHBQ	RbF		49
DHBQ	RbCl		49
DHBQ	RbBr		49
DHBQ	RbI		49

matrix	salt	$^{PS}Y_{calc}$	$^{PEG}Y_{calc}$
Dith	AgF	45	53
Dith	AgCl	40	53
Dith	AgBr	37	53
Dith	AgI	35	53
Dith	$AgNO_3$	27	57
Dith	CuCl	41	67
Dith	CuBr	40	67
Dith	CuI	38	67
Dith	$CuCl_2$	29	66
Dith	$Cu(NO_3)_2$	21	62
Dith	LiF	68	63
Dith	LiCl	76	63
Dith	LiBr	79	63
Dith	LiI	82	63
Dith	NaF	88	56
Dith	NaCl	95	56
Dith	NaBr	97	57
Dith	NaI	99	57
Dith	KF		47
Dith	KCl		48
Dith	KBr		48
Dith	KI		48
Dith	RbF		44
Dith	RbCl		44
Dith	RbBr		44
Dith	RbI		44

Supplementary Table 9. Laser fluence threshold predicted for PS ($^{PS}Y_{calc}$, in %) and for PEG ($^{PEG}Y_{calc}$, in %) for the 26 matrix/salt couples involving FA (left) or HABA (right). The second value into parentheses was determined experimentally. Color code: $Y_{calc} \leq 50\%$, green; $50\% < Y_{calc} \leq 75\%$, orange; $75\% < Y_{calc}$, red. Empty grey cells designate undetermined data (see text).

matrix	salt	$^{PS}Y_{calc}$	$^{PEG}Y_{calc}$
FA	AgF	31	47
FA	AgCl	31	47
FA	AgBr	31	47
FA	AgI	31 (28)	47
FA	$AgNO_3$	30	49
FA	CuCl	38	53
FA	CuBr	39	53
FA	CuI	40	53
FA	$CuCl_2$	26 (27)	52 (39)
FA	$Cu(NO_3)_2$	31	50
FA	LiF	53 (31)	51
FA	LiCl	79 (100)	52
FA	LiBr	86 (100)	52
FA	LiI	95 (100)	52
FA	NaF	80	49
FA	NaCl	100 (100)	49
FA	NaBr	106	49
FA	NaI	115 (100)	49
FA	KF		45
FA	KCl		45
FA	KBr		45
FA	KI		45
FA	RbF		44
FA	RbCl		44
FA	RbBr		44
FA	RbI		44

matrix	salt	$^{PS}Y_{calc}$	$^{PEG}Y_{calc}$
HABA	AgF	33	31
HABA	AgCl	33	31
HABA	AgBr	34	31
HABA	AgI	34	31
HABA	$AgNO_3$	35	24
HABA	CuCl	31	5
HABA	CuBr	33	5
HABA	CuI	35	5
HABA	$CuCl_2$	19	2
HABA	$Cu(NO_3)_2$	27	8
HABA	LiF	45	12
HABA	LiCl	48	13
HABA	LiBr	49 (42)	13 (24)
HABA	LiI	50	13
HABA	NaF	92 (100)	25
HABA	NaCl	95	25
HABA	NaBr	96	25
HABA	NaI	97	25
HABA	KF		42 (35)
HABA	KCl		43
HABA	KBr		43
HABA	KI		43
HABA	RbF		49
HABA	RbCl		49
HABA	RbBr		49
HABA	RbI		50

Supplementary Table 10. Laser fluence threshold predicted for PS ($^{PS}Y_{calc}$, in %) and for PEG ($^{PEG}Y_{calc}$, in %) for the 26 matrix/salt couples involving HCCA (left) or HPA (right). The second value into parentheses was determined experimentally. Color code: $Y_{calc} \leq 50\%$, green; $50\% < Y_{calc} \leq 75\%$, orange; $75\% < Y_{calc}$, red. Empty grey cells designate undetermined data (see text).

matrix	salt	$^{PS}Y_{calc}$	$^{PEG}Y_{calc}$
HCCA	AgF	22	49
HCCA	AgCl	24	49
HCCA	AgBr	27	49
HCCA	AgI	29	49
HCCA	$AgNO_3$	34	46
HCCA	CuCl	33	37
HCCA	CuBr	36	38
HCCA	CuI	39	38
HCCA	$CuCl_2$	21	35 (27)
HCCA	$Cu(NO_3)_2$	35	38
HCCA	LiF	46	41
HCCA	LiCl	41	41
HCCA	LiBr	39	41
HCCA	LiI	38 (25)	41
HCCA	NaF	37	46
HCCA	NaCl	33	46
HCCA	NaBr	32	46
HCCA	NaI	30	47
HCCA	KF		54
HCCA	KCl		54
HCCA	KBr		54
HCCA	KI		54
HCCA	RbF		57
HCCA	RbCl		57
HCCA	RbBr		57
HCCA	RbI		57

matrix	salt	$^{PS}Y_{calc}$	$^{PEG}Y_{calc}$
HPA	AgF	41	73
HPA	AgCl	40	73
HPA	AgBr	41	73
HPA	AgI	41	73
HPA	$AgNO_3$	41 (40)	83 (100)
HPA	CuCl	58	108
HPA	CuBr	60	108
HPA	CuI	61	108
HPA	$CuCl_2$	46 (45)	108
HPA	$Cu(NO_3)_2$	52	100 (100)
HPA	LiF	95 (100)	97 (100)
HPA	LiCl	51	98
HPA	LiBr	35	98
HPA	LiI	15	98
HPA	NaF	95	81
HPA	NaCl	50	81
HPA	NaBr	37	81
HPA	NaI	18	82
HPA	KF		58
HPA	KCl		59
HPA	KBr		59
HPA	KI		59
HPA	RbF		50
HPA	RbCl		50 (49)
HPA	RbBr		50
HPA	RbI		50

Supplementary Table 11. Laser fluence threshold predicted for PS (${}^{PS}Y_{calc}$, in %) and for PEG (${}^{PEG}Y_{calc}$, in %) for the 26 matrix/salt couples involving IAA (left) or MBT (right). The second value into parentheses was determined experimentally. Color code: $Y_{calc} \leq 50\%$, green; $50\% < Y_{calc} \leq 75\%$, orange; $75\% < Y_{calc}$, red. Empty grey cells designate undetermined data (see text).

matrix	salt	${}^{PS}Y_{calc}$	${}^{PEG}Y_{calc}$
IAA	AgF	32	70
IAA	AgCl	30	70
IAA	AgBr	30	70
IAA	AgI	29	70
IAA	$AgNO_3$	27 (26)	74 (100)
IAA	CuCl	39	85
IAA	CuBr	40	85
IAA	CuI	40	85
IAA	$CuCl_2$	27	84
IAA	$Cu(NO_3)_2$	29	81
IAA	LiF	48	80
IAA	LiCl	81	81
IAA	LiBr	89	81
IAA	LiI	101	81
IAA	NaF	86	73
IAA	NaCl	112	74
IAA	NaBr	119	74
IAA	NaI	130	74
IAA	KF		63
IAA	KCl		64
IAA	KBr		64
IAA	KI		64
IAA	RbF		59
IAA	RbCl		60
IAA	RbBr		60
IAA	RbI		60

matrix	salt	${}^{PS}Y_{calc}$	${}^{PEG}Y_{calc}$
MBT	AgF	39	63
MBT	AgCl	34	63
MBT	AgBr	31	63
MBT	AgI	29	63
MBT	$AgNO_3$	21	75 (100)
MBT	CuCl	46	108
MBT	CuBr	45	108
MBT	CuI	44	108
MBT	$CuCl_2$	35	109
MBT	$Cu(NO_3)_2$	27	98
MBT	LiF	14	95
MBT	LiCl	6	95
MBT	LiBr	3	95
MBT	LiI	0	95
MBT	NaF	26	74
MBT	NaCl	19	74
MBT	NaBr	17	74
MBT	NaI	15	74
MBT	KF		44
MBT	KCl		44
MBT	KBr		45
MBT	KI		45
MBT	RbF		33
MBT	RbCl		33
MBT	RbBr		33
MBT	RbI		33

Supplementary Table 12. Laser fluence threshold predicted for PS (${}^{PS}Y_{calc}$, in %) and for PEG (${}^{PEG}Y_{calc}$, in %) for the 26 matrix/salt couples involving NOR (left) or SA (right). The second value into parentheses was determined experimentally. Color code: $Y_{calc} \leq 50\%$, green; $50\% < Y_{calc} \leq 75\%$, orange; $75\% < Y_{calc}$, red. Empty grey cells designate undetermined data (see text).

matrix	salt	${}^{PS}Y_{calc}$	${}^{PEG}Y_{calc}$
NOR	AgF	34	59
NOR	AgCl	30	59
NOR	AgBr	27	59
NOR	AgI	25	59
NOR	$AgNO_3$	18	71
NOR	CuCl	42 (42)	103 (100)
NOR	CuBr	41	103
NOR	CuI	40	103
NOR	$CuCl_2$	30	104
NOR	$Cu(NO_3)_2$	23	93
NOR	LiF	16	90
NOR	LiCl	46	91
NOR	LiBr	54	91
NOR	LiI	65	91
NOR	NaF	44	70
NOR	NaCl	68	70
NOR	NaBr	74	70
NOR	NaI	84	70
NOR	KF		41
NOR	KCl		41
NOR	KBr		42
NOR	KI		42
NOR	RbF		30
NOR	RbCl		30
NOR	RbBr		30
NOR	RbI		31

matrix	salt	${}^{PS}Y_{calc}$	${}^{PEG}Y_{calc}$
SA	AgF	38	42
SA	AgCl	37	43
SA	AgBr	37	43
SA	AgI	38	43
SA	$AgNO_3$	37	40
SA	CuCl	39	31
SA	CuBr	40	31
SA	CuI	41	31
SA	$CuCl_2$	27	29
SA	$Cu(NO_3)_2$	32 (31)	31 (33)
SA	LiF	83	34
SA	LiCl	107	35
SA	LiBr	114	35
SA	LiI	123	35
SA	NaF	107	40
SA	NaCl	127	40
SA	NaBr	133	40
SA	NaI	141	40
SA	KF		48
SA	KCl		48
SA	KBr		48
SA	KI		48 (33)
SA	RbF		51
SA	RbCl		51
SA	RbBr		51
SA	RbI		51

Supplementary Table 13. Laser fluence threshold predicted for PS ($^{PS}Y_{calc}$, in %) and for PEG ($^{PEG}Y_{calc}$, in %) for the 26 matrix/salt couples involving THAP. The second value into parentheses was determined experimentally. Color code: $Y_{calc} \leq 50\%$, green; $50\% < Y_{calc} \leq 75\%$, orange; $75\% < Y_{calc}$, red. Empty grey cells designate undetermined data (see text).

matrix	salt	$^{PS}Y_{calc}$	$^{PEG}Y_{calc}$
THAP	AgF	40	72
THAP	AgCl	34	72
THAP	AgBr	32	72
THAP	AgI	29	72
THAP	$AgNO_3$	19	86
THAP	CuCl	47	121
THAP	CuBr	45	121
THAP	CuI	43	121
THAP	$CuCl_2$	35	122
THAP	$Cu(NO_3)_2$	25	111
THAP	LiF	25	107
THAP	LiCl	22	107
THAP	LiBr	21	107
THAP	LiI	19	107
THAP	NaF	42	84
THAP	NaCl	39	84
THAP	NaBr	38 (43)	84 (51)
THAP	NaI	37	84
THAP	KF		51
THAP	KCl		51
THAP	KBr		52
THAP	KI		52
THAP	RbF		39
THAP	RbCl		39
THAP	RbBr		39
THAP	RbI		39

4 MALDI-MS of PEO-*b*-PS copolymer

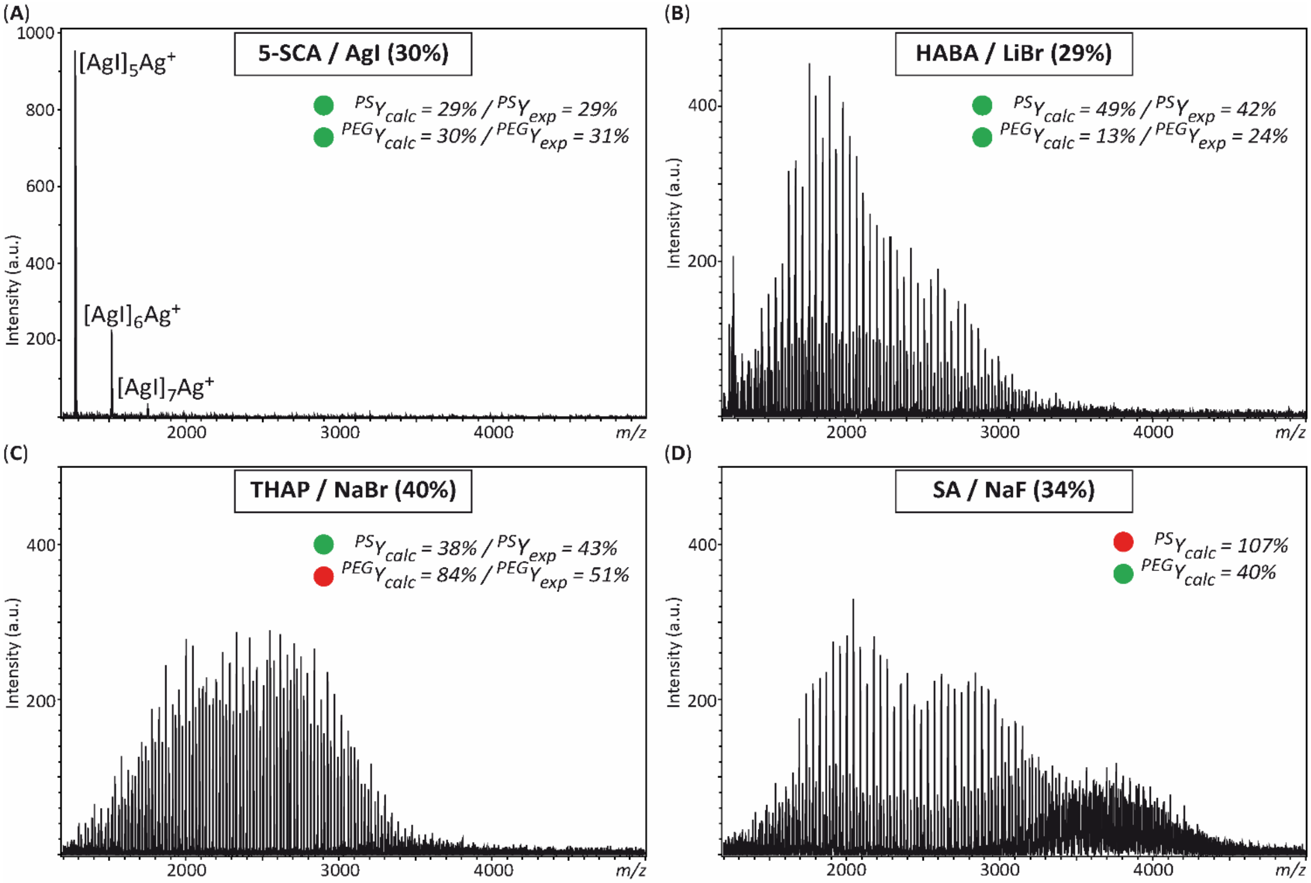

Supplementary Figure 2. MALDI mass spectra recorded for the PEO-*b*-PS copolymer when using (A) 5-CSA/AgI, (B) HABA/LiBr, (C) THAP/NaBr, or (D) SA/NaF as the matrix/salt couple, with the employed laser fluence into parenthesis. Inset: Predicted (Y_{calc}) or experimental (Y_{exp}) laser fluence when these experimental conditions are employed for PS and PEG homopolymers, using the same color code as in Supplementary Tables 4-13 to qualify laser fluence requirement (≤50%, in green; 51-75%, in orange; > 75%, in red).

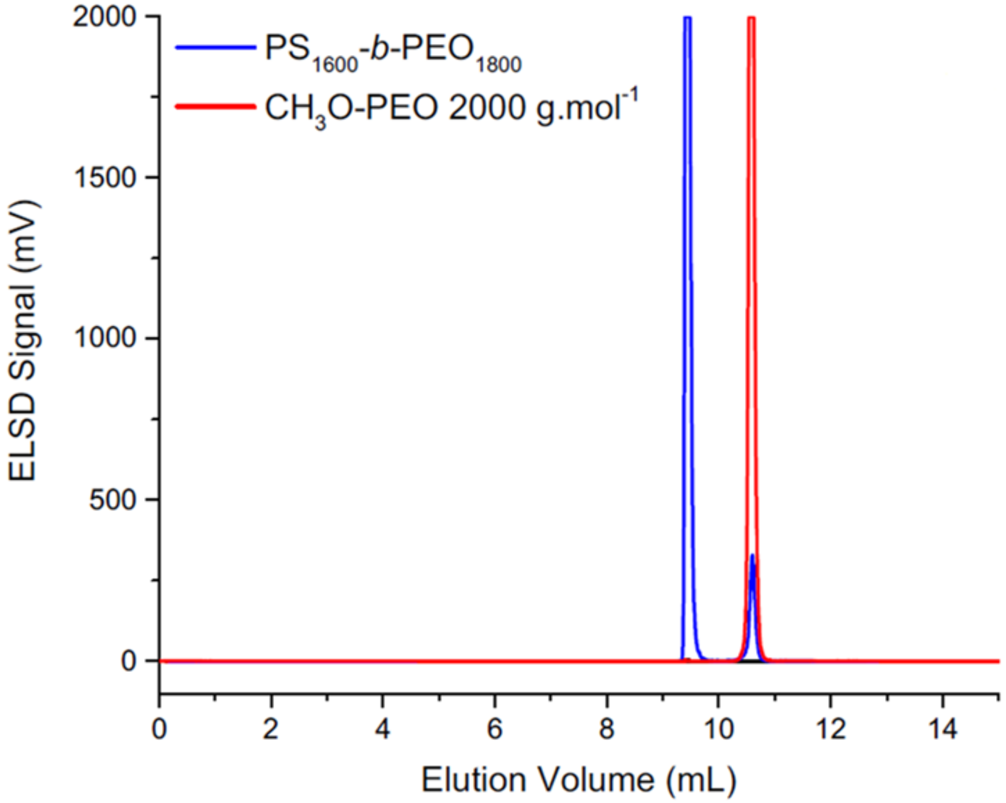

Supplementary Figure 3. Liquid chromatography at limit conditions of desorption (LC-LCD) of the PEO_{1800}-*b*-PS_{1600} copolymer (in blue), showing that this sample also contains residual PEO observed at the same elution volume as a CH_3O-PEO_{2000} standard (in red). Experimental conditions: column: Kromasil SiOH 300 x 7.8 mm (60 Å, 10 µm) at 30°C; eluent: dimethylformamide/1-chlorobutane (40/60, w/w) at a 1 mL min^{-1} flow rate; barrier 1: 100% 1-chlorobutane, 1 mL injected at t = 0 min; barrier 2: dimethylformamide/1-chlorobutane (30/70, w/w), 1 mL injected at t = 2 min; sample (10 mg in 4 mL of the eluent), 50 µL injected at t = 3.10 min; detection: evaporative light scattering detection (ELSD) at 70°C using a nebulization gas (N_2) flow rate of 60 psi.

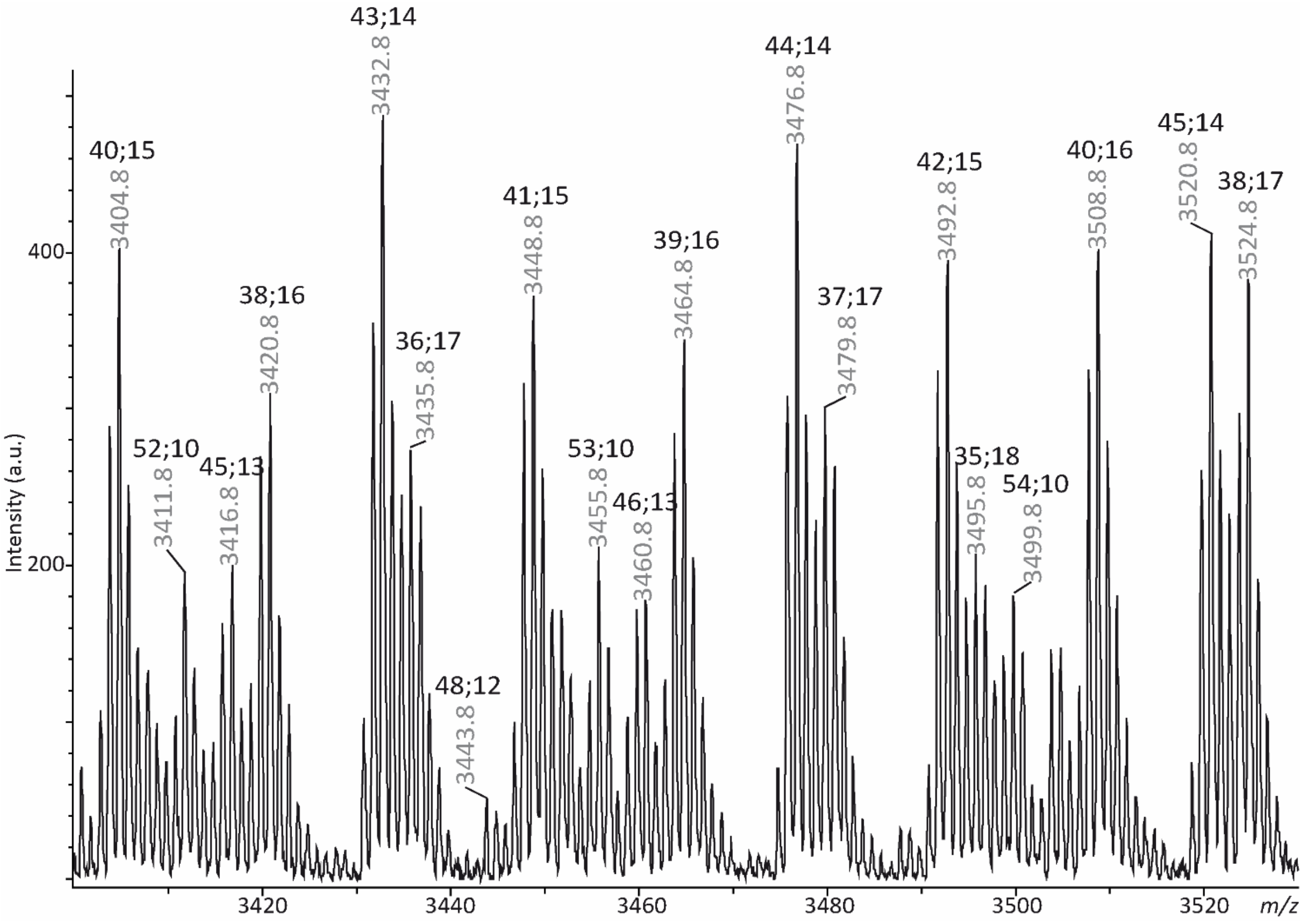

Supplementary Figure 4. Peak assignments in the 3400-3530 *m/z* range of the MALDI mass spectrum recorded for PEO-*b*-PS block copolymer when using 2,6-DHB/NaF: *m/z* values measured for sodium adducts (in grey) and n;m composition of each PEO_n-*b*-PS_m oligomer are consistent with the methylpropyl/H set of end-groups indicated by the supplier.

5 Complete list of authors for Gaussian software

Frisch, M. J.; Trucks, G. W.; Schlegel, H. B.; Scuseria, G. E.; Robb, M. A.; Cheeseman, J. R.; Montgomery Jr, J. A.; Vreven, T.; Kudin, K. N.; Burant, J. C.; Millam, J. M.; Iyengar, S. S.; Tomasi, J.; Barone, V.; Mennucci, B.; Cossi, M.; Scalmani, G.; Rega, N.; Petersson, G. A.; Nakatsuji, H.; Hada, M.; Ehara, M.; Toyota, K.; Fukuda, R.; Hasegawa, J.; Ishida, M.; Nakajima, T.; Honda, Y.; Kitao, O.; Nakai, H.; Klene, M.; Li, X.; Knox, J. E.; Hratchian, H. P.; Cross, J. B.; Adamo, C.; Jaramillo, J.; Gomperts, R.; Stratmann, R. E.; Yazyev, O.; Austin, A. J.; Cammi, R.; Pomelli, C.; Ochterski, J. W.; Ayala, P. Y.; Morokuma, K.; Voth, G. A.; Salvador, P.; Dannenberg, J. J.; Zakrzewski, V. G.; Dapprich, S.; Daniels, A. D.; Strain, M. C.; Farkas, O.; Malick, D. K.; Rabuck, A. D.; Raghavachari, K.; Foresman, J. B.; Ortiz, J. V.; Cui, Q.; Baboul, A. G.; Clifford, S.; Ciolowski, J.; Stefanov, B. B.; Liu, G.; Liashenko, A.; Piskorz, P.; Komaromi, I.; Martin, R. L.; Fox, D. J.; Keith, T.; Al-Laham, M. A.; Peng, C. Y.; Nanayakkara, A.; Challacombe, M.; Gill, P. M. W.; Johnson, B.; Chen, W.; Wong, M. W.; Gonzalez, C.; Pople, J. A. Gaussian 03, Revision C.02, Wallingford: 2004.
